# A Literature Overview of Virtual Reality (VR) in Treatment of Psychiatric Disorders: Recent Advances and Limitations

**DOI:** 10.3389/fpsyt.2019.00505

**Published:** 2019-07-19

**Authors:** Mi Jin Park, Dong Jun Kim, Unjoo Lee, Eun Jin Na, Hong Jin Jeon

**Affiliations:** ^1^Department of Psychiatry, Depression Center, Samsung Medical Center, Sungkyunkwan University School of Medicine, Seoul, South Korea; ^2^Department of Health Sciences & Technology, Department of Medical Device Management & Research, and Department of Clinical Research Design & Evaluation, Samsung Advanced Institute for Health Sciences & Technology (SAIHST), Sungkyunkwan University, Seoul, South Korea; ^3^Department of Electronic Engineering, Hallym University, Kangwon, South Korea

**Keywords:** virtual reality, psychiatric treatment, psychiatric disorders, dementia, motion sickness

## Abstract

In this paper, we conduct a literature survey on various virtual reality (VR) treatments in psychiatry. We collected 36 studies that used VR to provide clinical trials or therapies for patients with psychiatric disorders. In order to gain a better understanding of the management of pain and stress, we first investigate VR applications for patients to alleviate pain and stress during immersive activities in a virtual environment. VR exposure therapies are particularly effective for anxiety, provoking realistic reactions to feared stimuli. On top of that, exposure therapies with simulated images are beneficial for patients with psychiatric disorders such as phobia and posttraumatic stress disorder (PTSD). Moreover, VR environments have shown the possibility of changing depression, cognition, even social functions. We review empirical evidence from VR-based treatments on psychiatric illnesses such as dementia, mild cognitive impairment (MCI), schizophrenia and autism. Through cognitive training and social skill training, rehabilitation through VR therapies helps patients to improve their quality of life. Recent advances in VR technology also demonstrate potential abilities to address cognitive and functional impairments in dementia. In terms of the different types of VR systems, we discuss the feasibility of the technology within different stages of dementia as well as the methodological limitations. Although there is room for improvement, its widespread adoption in psychiatry is yet to occur due to technical drawbacks such as motion sickness and dry eyes, as well as user issues such as preoccupation and addiction. However, it is worth mentioning that VR systems relatively easily deliver virtual environments with well-controlled sensory stimuli. In the future, VR systems may become an innovative clinical tool for patients with specific psychiatric symptoms.

## Introduction

Virtual reality (VR) is defined as a computer-generated simulation, such as a set of images and sounds that represents a real place or situation, that can be interacted with, in a seemingly real or physical way by a person using special electronic equipment. It can transmit visual, auditory, and various sensations to users through a headset to make them feel as if they are in a virtual or imagined environment (1).

The concept of VR was introduced in the 1950s, and the maturity of VR for entertainment is now evident. Currently, more than 230 companies are producing various products related to VR and performing research and development, including global companies such as Samsung Electronics, Apple, Facebook, Amazon, and Microsoft. VR systems consist of VR headsets, a computer, and video. Recently, chairs, gloves, and sensors have been added. VR headsets refer to head-mounted goggles. They are equipped with a speaker or headphones. VR systems that include the transmission of vibrations and other sensations to the user through a game controller, gloves or chairs are known as haptic feedback systems (2). This tactility is advantageous as the sense of presence can be improved by actually sensing the shock or vibration to the user in the medical field, video games, and military training. A 4D (four-dimensional) system of VR refers to a VR system with a motion chair that enhances reality for users with integrated movement according to the content of the VR video. Depending on the type of system and programing, the user may interact with the environment from a first- or third-person’s point of view. In the case of the latter, the user can move around a virtual representation of themselves, called an ‘avatar’ (3).

In medical fields, multidisciplinary research has tried to apply VR systems to domains of diagnosis, treatment, and so on. Especially, in psychiatry, traditional tools of treatment have mainly been limited to interpersonal psychotherapy and medication. However, VR can provide various types of stimulation (4). Intuitively, it helps in relieving pain, stress, and anxiety in an imagined space, and VR makes it possible to provide efficient educational and psychological training without causing harm to patients (5). It therefore has the possibility of changing PTSD, phobia, anxiety, depression, cognition, and social functions in patients with psychiatric illnesses.

Indeed, over the past few decades, therapeutic virtual reality (VR) has emerged as a successful solution for a wide range of psychiatric disorders. In the 1990s, Rothbaum et al. conducted the first study in the field of psychiatry to investigate the efficacy of VR focusing one treating acrophobia in college students and found that VR is successful in reducing their fear of heights (6). The early studies established the efficacy of VR exposure therapy for a number of anxiety and related disorders. For example, VR exposure therapies have shown benefits for patients with a specific phobia or posttraumatic stress disorder (PTSD) by the extinction of traumatic experiences through their repetitive exposures, and the extinction of pain by pulling the patients focus away from painful conditions. The broad reach of VR has enabled its use in the evaluation and rehabilitation of patients with schizophrenia and autism through improvements of their social activities. The reports included in this review, show that VR is also an efficacious way for Amnestic MCI (mild cognitive impairment) and early to moderate Alzheimer’s disease through cognitive reserve and training.

VR-based treatment currently faces hurdles preventing its wide use as a real tool in psychiatry practice, such as motion sickness and dry eyes as well as user issues such as preoccupation and addiction. However, VR systems can deliver and confront virtual environments with well-controlled sensory stimuli. With a review of the current utilization of VR in the field of psychiatry, we highlight both the benefits and limitations of VR use, as it is just beginning to be applied as a new modality in psychiatry. We have tried to describe the evidence of the utility of VR in psychiatric conditions and the types of procedures followed in those studies

## Methods and Results

To identify ‘virtual reality’ in the field of psychiatry, a PubMed literature search was performed which included articles published prior to 31 December 2018, and which included the terms “virtual reality” and “psychiatry” and “treatment” or “therapy.” We then categorized them as articles related to “posttraumatic Stress Disorders (PTSD),” “phobia,” “anxiety disorders,” “schizophrenia,” “autism,” “dementia and mild cognitive impairment (MCI),” and “pain and stress.” Additional relevant articles were found through a manual bibliographic search. Eligibility criteria were: 1) articles in English; 2) human studies; and 3) articles focused on virtual reality and psychiatric disorders. We collect 36 studies that made use of VR to provide various types of stimulations for patients with psychiatric problems (**Table 1**).

**Table 1:** Clinical trials or therapies with virtual reality (VR) in psychiatry.

Author and date of publication	Subjects	Design	Method	Conclusions
Doniger et al., 2018 (7)	Middle-aged adults with Alzheimer’s disease family history (*n* = 125)	RCT	VR cognitive-motor training, 45 min, twice/week for 12 weeks	Increased cognitive function
Reger et al., 2019 (8)	Active duty soldiers with PTSD (*n* = 108)	Observational	Randomization to exposure *via* 10 sessions of prolonged exposure or VRE or 5-week minimal attention waitlist	No group differences in average or peak subjective distress during exposure therapy
Flores et al., 2018 (9)	Two patients with spinal cord injury with psychiatric symptoms	Case report	4 VR DBT sessions for Patient 1, 2 VR DBT sessions for Patient 2	Reductions in negative emotions for Patient 1, mixed results for Patient 2
Peskin et al., 2018 (10)	Men and women with World trade center-related PTSD (*n* = 25)	RCT	100 mg D-cycloserine versus placebo augmented VRE sessions for 12 weeks	Temporal relationship between posttraumatic and depressive symptoms. during VRE
Du Sert et al., 2018 (11)	Schizophrenia patients with refractory AVH (*n* = 19)	RCT	A 7-week phase-II, randomized, partial cross-over trial	Significant improvements in severity of auditory visual hallucination, depressive symptoms and quality of life
Pot-Kolder et.al., 2018 (12)	Patients with a psychotic disorder and paranoid ideation (*n* = 116)	RCT	VR-CBT with treatment as usual, 1 h long, 16 individual session versus treatment as usual	Significant reduction in momentary paranoid ideation and anxiety
Gold et al., 2018 (13)	Child and adolescent patients (*n* = 143)	RCT	Totally 5 min long VR game with standard of care versus standard of care only	Significant reduction in acute procedural pain and anxiety
Gomez et al., 2017(14)	21-year-old Latino male patient with burn injury	Observational	Immersive VR enhanced DBT mindfulness skills training, 4 sessions for 1 month	Increased positive emotions and decreased negative emotions
Ryu et al., 2017 (15)	Children scheduled for elective surgery (*n* = 69)	RCT	Preoperative VR tour of the operating theatre, 4 min video	Lower scores of the Yale Preoperative Anxiety Scale, Induction Compliance Checklist, and Procedural Behavior Rating Scale
Eijlers et al. 2017 (16)	Children undergoing elective day care surgery (*n* = 200)	RCT	Preoperative VRE intervention, 15 min	Diminished preoperative anxiety, postoperative pain, and the use of postoperative analgesics
Beidel et al., 2017 (17)	Veterans and active duty soldiers with combat-related PTSD (*n* = 92)	RCT	VRET plus group treatment versus VRET with psychoeducation control	Decrease on PTSD scale for both group and decrease in social isolation for VRET plus group treatment
Ferrer-Garcia et al., 2017 (18)	Patients with bulimia nervosa and binge eating disorder (*n* = 64)	Case control	Two second-level treatment condition: VR- Cue Exposure Therapy or additional CBT	More proportion of achievement abstinence from binge eating or purging episodes
Blume et al., 2017 (19)	Children with ADHD (*n* = 90)	RCT	15 training sessions of either NIRS based NFT in VR, NIRS based NFT in 2D or biofeedback training in VR, 60–70 min for each session	NFT in VR is expected to yield greater effects than training in 2D
Shiban et al., 2017 (20)	29 patients with aviophobia	RCT	VRE treatment either with or without diaphragmatic breathing	Higher tendency to effectively overcome the fear of flying in VR with diaphragmatic breathing
Bouchard et al., 2017 (21)	Patients with social anxiety disorder (*N* = 59)	RCT	14 weekly sessions for VR exposure or *in vivo* exposure or waiting list	Improvement in both CBT groups, more effective in VRE
Reger et al., 2016 (22)	Active-duty soldiers (*n* = 162)	RCT	Randomization to 10 sessions of Prolonged exposure, VRE, or a minimal attention waitlist	Significant reductions in PTSD symptoms in Prolonged exposure and VRE groups
Norrholm et al., 2016 (23)	Participants met criteria for PTSD (*n* = 50)	RCT	6 weeks of VRE therapy combined with d-cycloserine, alprazolam, or placebo	In the d-cycloserine group, elevated startle *before* VR therapy predicted better outcome
Son et al., 2015 (24)	Alcohol dependent subjects (*n* = 12)	Case control study	10 sessions of VRET, consisted of 3 steps: relaxation, presentation of a high risk situation, and aversive situation	Decreased metabolism in the basal ganglia after VRET (PET shows)
Jahani Shoorab et.al., 2015 (25)	Primiparous parturient women having labor (*n* = 30)	RCT	Randomization to VR with standard care group and only standard care group	Decreased pain during the episiotomy repair When use of VR with local anesthesia
Rothbaum et al., 2014 (26)	Iraq and Afghanistan war veterans with PTSD (*n* = 156)	RCT	An introductory session and five sessions of VRE augmented with d-cycloserine or alprazolam or placebo	Significantly improved PTSD symptoms from pre- to posttreatment across all conditions
Marco et al., 2013 (27)	Participants diagnosed with eating disorders (*n* = 34)	Case control study	15 CBT group sessions and 8 individual psychotherapy sessions with VR	Improved body image and this improvement was maintained at the one-year follow-up
Pallavicini et al., 2013 (28)	Undergraduate students (*n* = 39)	Case control study	Same experience was offered using test, audio, video, and VR	VR was less effective than other procedures in eliciting stressor responses
Diemer et al., 2013 (29)	Patients with arachnophobia (*n* = 58)	RCT	A single dose of quetiapine XR or placebo prior to a VR	Effect of VR challenge on behavioral avoidance, psychophysiological reaction
Malbos et al., 2013 (30)	Agoraphobic participants (*n* = 18)	Case control study	VRET only and VRET with cognitive therapy	The isolated effects of VRET did not seem to be less than the effects of VRET with cognitive therapy
McLay et al. 2012 (31)	Active duty service members with PTSD (*n* = 20)	Observational	Open-label, single-group VRET	Reduction in PTSD symptoms, improvement in PTSD, depression and anxiety
Culbertson et al., 2012 (32)	Healthy treatment-seeking cigarette smokers (*n* = 11)	RCT	Randomization to CBT plus either smoking-VR Cue Exposure Therapy or placebo-VR Cue Exposure Therapy	Higher quit rate, smoking fewer cigarettes per day
Park et al., 2011 (33)	Inpatients with schizophrenia (*n* = 91)	RCT	Comparison social skills training using VR role playing to social skills training using traditional role playing, over 10 semiweekly sessions for 5 weeks	Improved more in conversational skills and assertiveness
McLay et al., 2011 (34)	Active duty military personnel with combat-related PTSD (*n* = 10)	RCT	Randomization to VR-graded exposure therapy or treatment as usual	Higher number of improvements reported, more improvement on the CAPS score
St-Jacques et al., 2010 (35)	Agoraphobic participants (*n* = 31)	RCT	Randomization to *in vivo* exposure alone or in virtual reality-based exposure	VR did not increase motivation toward psychotherapy
Gerardi et al., 2008 (36)	A 29-year-old veteran	Case report	90-min individual session, once weekly over 4 weeks	Decreased rating scale scores (CAPS, PTSD Symptom Scale Self-Report)
Difede et al., 2007 (37)	Male disaster workers with PTSD (*n* = 21)	Case control study	Assignment to a VR treatment or a waitlist control	Significant decline in CAPS scores
Walshe et al., 2003 (38)	Subjects with simple phobia/accident phobia (*n* = 14)	Observational	An open study, computer games and virtual reality therapy, 12 1-h sessions	Significant post treatment reductions on all measures
Rothbaum et al., 2001 (39)	Male Vietnam combat veterans with PTSD (*n* = 10)	Observational	Open clinical trial, 8 to 16 sessions, 2 virtual environments	Significant reduction from baseline in symptoms
Rothbaum et al., 2000 (40)	Patients with fear of flying (*n* = 49)	Case control study	Randomization to VRE therapy, standard exposure therapy, or a wait-list control, 4 sessions of exposure out of 8 sessions	VRE and standard exposure both superior to wait-list
Rothbaum et al., 1999 (41)	A Vietnam combat veteran with PTSD	Observational	VRE, 2 virtual environments	Decrease on CAPS and self-rated PTSD
Rothbaum et al., 1996 (42)	A 42-year-old female with a debilitating fear and avoidance of flying	Case report	Anxiety management techniques and the VRE	All self-report measures of fear and avoidance of flying decreased

RCT, randomized controlled trial; VR, virtual reality; VRE, virtual reality exposure; VRET, virtual reality exposure therapy; DBT, dialectical behavior therapy; PTSD, posttraumatic stress disorder; CBT, cognitive-behavioural therapy; NIRS, near-infrared spectroscopy; CAPS, clinician administered PTSD scale; NFT, neurofeedback training.

## Discussion

### Treatment of Posttraumatic Stress Disorders (PTSD)

Posttraumatic Stress Disorder (PTSD) is a psychological reaction that occurs after experiencing stress that has caused life-threatening extreme mental trauma (42). An individual’s quality of life is greatly reduced by re-experiencing the situation with awakening, anxiety, agitation, and insomnia symptoms. Among PTSD, many VR studies have been focused on veterans who have been exposed to battles in Iraq and Afghanistan, to alleviate their trauma (43), reduce suicidal ideation (44), decrease depression and anger (16), and to improve their PTSD (30). Discharged soldiers can have destructive behaviors to both themselves and others as a result of rage and depression caused by PTSD. However, they can learn to solve these situations in a safe and well-controlled environment called VR. Since the key in the emotion-processing theory (EPT) is to expose and modify their unique fear structure, the virtual environment is ideal in the sense of its flexibility and customization (45). As they are exposed to sources of their disorder, they decrease the feelings of fear and anxiety in the form of VR-based habituation therapy. Dr. Rothbaum at the Emory University Hospital provided a randomized, double-blind, six-session VR exposure treatment in 156 patients diagnosed with PTSD among discharged soldiers returning from the Iraq and Afghan wars (26). The study concluded that VR treatment was associated with the reduction in PTSD diagnoses and symptoms in Iraq and Afghanistan veterans. Another study suggested that VR with skin conductance reactivity is a diagnostic tool for PTSD as well as a treatment (47). Veterans with PTSD displayed larger skin conductance reactivity across VR combat events, but not for non-combat VR events. The VR exposure therapy system, “Bravemind,” is currently distributed to over 50 sites, including VA hospitals, military bases, and university centers to provide relief from PTSD for soldiers (48).

### Anxiety Disorders and Specific Phobia

These days, some VR systems create highly immersive experiences using more invasive devices such as Head mounted Display (HMDs). A new generation of realistic simulation can therefore serve as a promising assessment and therapy for erroneous anxiety-provoking thinking. In general, these symptoms are viewed as serious conditions where patients worry about something fearful excessively and persistently. Reproducing the traditional exposure interventions in VR, Maples-Keller et al. reviewed several case studies of social anxiety disorders and generalized anxiety disorders (49). This is merely the beginning of an explosion in potential provided by ever increasing sophisticated technology. It worth noting that the effectiveness of applying VR in this domain is also quantitatively being analyzed (50).

More specifically, phobia is a type of anxiety disorder characterized by marked and persistent fears that are cued by the presence or anticipation of specific objects or situations with a desire to avoid that condition due to high levels of fear and discomfort (43). Phobia includes acrophobia, flight phobia, phobias for insects or animals, and so on. Exposure therapy in VR is helpful because we can deal with such specific phobias in a virtual world, and it can be cost-effectively performed (29). In VR, patients with phobia can reproduce the situation they actually feel fearful of and face it themselves. Repeated use of VR increases the threshold of anxiety and makes it less insensitive, resulting in the reduced incidence of actual situations. Initially, VR graded exposure therapy was found to be successful in reducing fear of spiders (51, 52), social phobia (53), and flight phobia (3, 54) after applying it to a small number of subjects. A self-training program with mobile VR individuals with acrophobia has been safely and successfully applied to reduce fear of heights (55). It can be safely applied at home and at the hospital. It can be easily interrupted or repeated depending on the situation. VR can reduce the degree of anxiety by exposing the patient to a virtual dental care scenario in an incremental manner (56, 57). Similarly, a pilot study has applied a VR headset as a fear reduction tool and pain distraction for fear of needles, where 94.1% of pediatric subjects reported an improvement after using VR during immunization (58). Recently, VR with repetitive transcranial magnetic stimulation (rTMS) over the prefrontal cortex has been applied in participants with spider phobias. It diminished activation in the left inferior frontal gyrus in functional near-infrared spectroscopy (fNIRS) during an emotional Stroop paradigm (59).

### Schizophrenia

Patients with schizophrenia show anhedonia, social withdrawal, and a blunted affect, which can lead to rumination and isolation. While exposure therapy in anxiety-related disorders uses VR as a simulation tool, the so-called avatar therapy for negative symptoms of schizophrenia focuses on interactive VR. In a computer-generated virtual world, VR users are no longer simply external observers, but active participants. It is one of the key variables in understanding social environments that need to be controlled, and thus provides exciting applications to research and treatment (60). For example, interactive VR therapy has shown benefits in social skills such as role-playing (33), memory function (61), medication management skills (62), job interviews (63), and vocational training (64). Recent VR-based cognitive rehabilitation programs also manage positive symptoms in schizophrenia such as auditory verbal hallucinations (11).

### Autism

Autism is characterized by a state of being trapped in one’s own world. It is a childhood developmental disability. Children with autism do not interact with others. They do not have emotional ties. VR approaches for rehabilitation in autism tend to create virtual environments integrated with other equipment, facilitating cognitive processes of training such as concentration and other functional skills in everyday life. The University of Texas has developed a training program to assist in the social skills training of autistic children (65). It uses brain imaging and Electroencephalography (EEG) monitoring. It also uses avatars to put children in situations such as job interviews and meetings. They practice reading social signals and expressing socially appropriate behaviors. After completion of the program, the activity of the brain area associated with social understanding was found to be increased in participants’ brain image. Smith and colleagues at the Northwestern University Psychiatry Department have reported that young adults diagnosed with autism spectrum have a higher job search rate than the comparative group at six months after receiving job interviews through VR (66). For the purpose of training outdoor activity, individuals with Autism spectrum disorder were placed in a three-dimensional city and given a set of tasks that involved taking buses through a game. A statistically significant increase in measures of knowledge of the process of riding a bus, a reduction in the electro-dermal activity, and a high success rate in their application were found (67).

### Dementia and Mild Cognitive Impairment (MCI)

Lessons from the Nun study have revealed that cognitive reserve and training are also important in preventing Alzheimer’s disease (68). Dementia is a broad term describing such disorders of the brain that progress over time. Basically, in evaluating cognitive dysfunction and detecting MCI, VR has been applied and has exhibited very high accuracy. Cushman et al. (69) have investigated navigational impairment of early Alzheimer’s disease, using both real-world and laptop PC based virtual environments in the same subjects; 35 young normal controls, 26 older normal controls, 12 patients with mild cognitive impairment and 14 patients with early Alzheimer's disease (AD). It was found that virtual environment testing provides a valid assessment of navigational skills for aging and Alzheimer’s disease (69). Also, there is a systematic review that presented a status of VR applications for diagnostic assessment and cognitive training in Alzheimer’s disease and MCI. Both semi-immersive and fully-immersive VR technology can be feasible amongst individuals living within the earlier stages of dementia outside of a hospital environment (70). While much of the VR studies appear to focus on the treatment of anxiety or phobias, the population of VR applications is underdeveloped. Even though sample sizes are limited, VR-based cognitive training has shown benefits for episodic memory in Amnestic MCI and early to moderate Alzheimer’s disease (71). Moreover, Moyle el al., explored the feasibility of VR in individuals with a range of cognitive impairments from mild to more severe stages of dementia (72). VR was perceived to have a positive effect on people with dementia, although a greater level of fear and anxiety during VR were experienced compared to those in the normative sample (72). Some individuals in the earlier stages of dementia experienced boredom, and VR technology was also found to increase fear and anxiety in one study. However, it is perhaps not surprising that recent advances in VR rehabilitation applications keep pointing to the feasibility of VR training in healthy elderly persons as well as in pathological populations (73).

### Stress and Pain Alleviation

Stress and pain have deleterious effects on the mind and body. In order to decrease one’s attention available for conscious pain processing, VR usage for stress and pain alleviation typically provides simple forms of distraction (e.g., watching videos or playing video games). Although the physical mechanisms are not well understood, the patients focus moves away from the conscious attention on the stressful and painful condition during the occupational activity (74). While patients can learn pain-management techniques as mindfulness, several experimental results suggest that VR techniques has actual benefits for subjective pain reduction (75). For example, Oculus Rift uses DEEP, a meditation application to help users breathe deeply. The application works through a band surrounding the chest to measure breathing. In another pilot study, 44 participants attended a mindfulness conference putting on an Oculus Rift DK2 VR helmet and floated down a calm 3D computer-generated virtual river while listening to digitized DBT mindfulness skills training instructions. Participants reported significantly less sadness, anger, and anxiety but more relaxation (76). Dr. Spiegel’s team at the Cedars-Sinai Hospital has given chronic patients the opportunity to get out of the hospital through VR and to enjoy the natural scenery. This could reduce a patient’s stress and shorten hospital stays (77). Relaxation and meditation in various VR applications have become increasingly widespread for treating patients at home or in hospitals (78).

Studies at the University of Barcelona have shown that applying VR to depressed patients can reduce the severity of their depression and self-degradation and increase satisfaction (79). By limiting distractions from the real world and increasing the sense of presence, VR may facilitate mindfulness practice as well.

### Limitations

Clearly, exposure to VR applications may result in significant discomfort for the majority of people, with symptoms of motion sickness including eye fatigue, headaches, nausea, and sweating (80, 81). VR Sickness is different from common motion sickness because motion sickness is caused by visual perception of self-motion while VR sickness does not require actual movement. A conflict between accommodation and vergence depth cues on stereoscopic displays is a significant cause of visual discomfort from VR (82). Dry eyes due to an overheated display in an enclosed space and retinal damage due to blue light are also concerns. As shown in this review, only a few large-sized and well-designed studies have been conducted in psychiatry with VR.

VR is developing to improve real-life adaptation of patients with psychiatric problems. However, patients may become preoccupied or addicted to the VR environment, similar to internet game addiction. If patients with schizophrenia have impairment on reality testing, they may have delusional thinking in the VR environment. Doctor-patient relationships and careful education before using VR are mandatory before applying VR treatments in psychiatric patients. In the near future, a guideline to apply VR treatments to patients with psychiatric illnesses should be established. VR will play a role as an alternative option for psychiatrists to use in supporting psychiatric assessments and treatments in patients.

## Conclusion

Many studies and clinical trials have used VR as a simulation, interaction, and distraction tool for patients with psychiatric illnesses such as PTSD, anxiety, specific phobia, schizophrenia, autism, dementia, and heavy stress. VR environments show the possibility of changing their anxiety, depression, cognition, and social functions by effectively exposing them sources of fear, presenting interactive virtual environments of cognitive-behavioral approaches, and contributing to other rehabilitation applications.

In practice, patients with a psychiatric diagnosis such as depression, bipolar disorder, anxiety disorder, schizophrenia, and even alcohol use disorder share common characteristics such as anxiety, avoidance, and poor insight to their illnesses. Modern VR systems can deliver an ideal place where one can confront the problem which needs to be overcome, not only through talking with doctors, but also through virtual environments with well-controlled sensory stimuli. This may produce cognitive and behavioral changes in patients with psychiatric disorders including autism and dementia. They also have benefits in reducing chronic pain and intensive stress. However, VR needs to overcome technical hurdles such as motion sickness and dry eyes, as well as user hurdles such as preoccupation and addiction.

## Author Contributions

All authors listed have made substantial, direct, and intellectual contribution to the work and approved it for publication.

## Funding

This work was supported by the Original Technology Research Program for Brain Science through the National Research Foundation of Korea (NRF) funded by the Ministry of Science and ICT (No. NRF-2016M3C7A1947307; PI HJJ), and by the Bio & Medical Technology Development Program of the NRF funded by the Korean government, MSIT (No. NRF-2017M3A9F1027323; PI HJJ). They had no further role in study design; in the collection, analysis and interpretation of data; in the writing of the report; and in the decision to submit the paper for publication.

## Conflict of Interest Statement

The authors declare that the research was conducted in the absence of any commercial or financial relationships that could be construed as a potential conflict of interest.
